# Microporous and Mesoporous Carbon Nanofibers from Lignin by In Situ Sodium Templating for Supercapacitor Electrodes

**DOI:** 10.3390/polym18182203

**Published:** 2026-09-10

**Authors:** Linet Hernández-Gil, Zhu Peng Shen, Ainhoa Álvarez-Gómez, María Fernández-Álvarez, Juan P. Fernández Blázquez, Juan C. Cabanelas, Verónica San-Miguel, María B. Serrano

**Affiliations:** 1 Department of Materials Science and Engineering and Chemical Engineering (IAAB), Universidad Carlos III de Madrid, Avda. de la Universidad 30, 28911 Leganes, Spain; khernand@ing.uc3m.es (L.H.-G.); zshen@ing.uc3m.es (Z.P.S.); caba@ing.uc3m.es (J.C.C.); 2 Instituto de Cerámica y Vidrio (ICV), CSIC, Kelsen 5, 28049 Madrid, Spain; maria.fernandez@icv.csic.es; 3 IMDEA Materials Institute, C/Eric Kandel 2, 28906 Getafe, Spain; juanpedro.fernandez@imdea.org

**Keywords:** lignin, electrospun carbon nanofibers, hierarchical porosity, sodium templating, supercapacitors

## Abstract

Lignin-derived carbon nanofibers (LCF) with hierarchical micro–mesoporous architecture featuring unusually high mesopore preservation were fabricated by electrospinning. The process used a lignin/poly(ethylene oxide) precursor in an alkaline medium (sodium hydroxide, NaOH), followed by carbonization and selective HCl washing. During electrospinning, sodium species were incorporated from the alkaline spinning solution and, upon subsequent thermal treatment, converted in situ into sodium-containing domains that acted as transient porogens, enabling the development of a highly accessible pore network without conventional activation. The resulting free-standing carbon nanofiber mats exhibited a hierarchical micro–mesoporous architecture, in which 84% of the specific surface area arose from micropores accompanied by a well-defined 8–12 nm mesopore population that is expected to facilitate ion transport, maximizing the utilization of the microporous surface for charge storage. Raman analysis further revealed a defect-rich, edge-abundant sp^2^ carbon network (*I_D_*/*I_G_* = 1.05). As self-supported supercapacitor electrodes in 3 M potassium hydroxide (KOH), the materials delivered a specific capacitance of ≈275 F g^−1^ at 0.25 A g^−1^, retained ≈87% of this value at 2.5 A g^−1^, and exhibited outstanding cycling stability, with 117% capacitance retention and 99.9% coulombic efficiency after 10,000 charge–discharge cycles. This simple, low-cost, and low-waste strategy demonstrates that in situ sodium templating effectively engineers hierarchical pore architectures in lignin-derived carbon nanofibers without aggressive chemical activation, providing a sustainable platform for high-performance energy-storage electrodes.

## 1. Introduction

Carbon fibers (CFs) and carbon nanofibers (CNFs) have traditionally been produced from fossil-derived precursors such as polyacrylonitrile (PAN) and mesophase pitch owing to their unique combination of low density, high mechanical strength, and electrical conductivity. However, the high cost, finite availability, and environmental impact of these precursors have intensified the search for more sustainable alternatives. Biomass has therefore emerged as a promising renewable feedstock for producing functional carbon materials, offering a near-closed carbon cycle, reduced net CO_2_ emissions, and potential carbon sequestration [1,2,3]. Thermochemical treatments such as pyrolysis and activation can tailor their structure and surface chemistry, enabling precise control over porosity, specific surface area, and surface functionality. Combined with heteroatom doping or surface modification, these materials have demonstrated competitive performance in electrochemical energy storage systems [4], including supercapacitors [5] and batteries [6], where high surface accessibility and efficient charge transport are essential.

Beyond controlling pore structure, the surface chemistry of biomass-derived carbons can be tailored through heteroatom doping (e.g., N, O, S, P, B) to enhance their electronic and electrochemical properties [4,7]. Nitrogen is one of the most widely used dopants and is commonly introduced through nitrogen-rich precursors such as urea, melamine, or dicyandiamide [8,9]. However, the extensive decomposition of these precursors during carbonization severely limits nitrogen retention. Consequently, in lignin-based systems, urea often acts as a sacrificial, gas-releasing additive that promotes pore formation rather than as an effective nitrogen dopant [10].

Among lignocellulosic biomass components, lignin is the most abundant renewable source of aromatic carbon in nature. Its large-scale industrial availability, primarily as a by-product of pulping and biorefinery processes, makes it an attractive and inexpensive feedstock for producing high-value carbon materials, offering a more sustainable alternative to its conventional use as a low-value fuel for internal energy recovery [11]. Lignin is a highly crosslinked aromatic biopolymer composed of phenylpropanoid units, whose high carbon content makes it an excellent precursor for carbon materials. However, its intrinsic structural heterogeneity limits precise control over pore development, particularly within the micropore range (<2 nm), which plays a key role in adsorption and electrochemical charge storage [12]. In lignin-derived carbon nanofibers, electrochemical performance is governed by the interplay between precursor chemistry, fiber formation, and hierarchical pore development across multiple length scales, where sub-nanometer micropores provide charge-storage sites while mesopores facilitate efficient ion transport [11,13,14].

Lignin-derived carbon nanofibers (LCF) have emerged as promising sustainable carbon materials, combining the renewable nature of lignin with the one-dimensional morphology and high aspect ratio provided by electrospinning [14,15,16]. This technique enables the fabrication of continuous nanofibrous networks with controlled fiber diameter and architecture from a wide range of precursor formulations [17,18]. Upon carbonization, lignin naturally develops a predominantly microporous carbon structure through the release of volatile species. To further increase the specific surface area, physical or chemical activation is commonly employed, producing BET surface areas approaching 1000 m^2^ g^−1^. However, these treatments generally increase microporosity at the expense of the mesoporosity required for efficient ion transport [19]. Consequently, electrospun LCFs have attracted considerable interest as sustainable, low-cost electrodes for supercapacitors [20] and lithium- and sodium-ion batteries, offering an attractive alternative to conventional PAN- and pitch-derived carbon fibers.

Despite these advances, most reported strategies have focused on maximizing specific surface area through chemical activation, sacrificial additives, or templating strategies [21,22,23], rather than on engineering hierarchical pore architectures with controlled pore-size distribution and connectivity. These approaches have produced lignin-derived carbon nanofibers with exceptionally high BET surface areas. For example, microporous carbon nanofiber membranes with surface areas of 1197 m^2^ g^−1^ have been reported [24], while values as high as 2340 m^2^ g^−1^ have been achieved in O_2_/H_3_PO_4_-activated electrospun lignin carbon fibers [25] and 2370 m^2^ g^−1^ in activated PAN/lignin-derived carbon nanofiber mats [26]. Nevertheless, maximizing BET surface area alone does not necessarily guarantee superior electrochemical performance, since only the electrochemically accessible surface contributes effectively to charge storage. Indeed, electrochemical performance is governed by the accessibility and hierarchical organization of the pore network rather than by the total surface area [27]. In this context, an interconnected mesopore network can provide efficient transport pathways to the microporous charge-storage domains, whereas excessive activation often may reduce mesoporosity, electrical conductivity, and structural integrity in pursuit of higher BET surface areas [28,29]. These limitations are further amplified in electrospun non-woven mats, where the large inter-fiber voids may reduce the volumetric efficiency of the electrode despite its high specific surface area [30]. Therefore, engineering hierarchical pore architectures that maximize accessible surface area rather than total BET surface area has emerged as a key design criterion for high-performance porous carbon electrodes [29,31].

Conventional activation strategies, typically based on mixing carbon precursors with porogenic agents prior to high-temperature treatment, increase microporous surface area by chemically etching the carbon framework [7]. However, this approach relies on external activating agents consuming carbon and provides limited control over pore hierarchy and surface chemistry. More recently, electrospun lignin-derived carbon nanofibers prepared from alkaline spinning solutions have demonstrated that sodium-containing phases generated during carbonization can be selectively removed by dilute acid washing to develop porosity in situ [32]. Although this strategy represents an elegant alternative to conventional activation, previous studies have primarily aimed at increasing pore development, whereas simultaneous control of hierarchical pore architecture, mesopore preservation, and surface chemistry has received comparatively little attention.

Early work by the Titirici group demonstrated the potential of electrospun lignin-derived carbon nanofibers as free-standing supercapacitor electrodes, using finely dispersed alkali-derived domains to tailor microporosity and achieving about 180 F g^−1^ with a comparatively moderate surface area (~670 m^2^ g^−1^) [33]. Subsequent work showed that densification of these fibrous mats could substantially improve volumetric performance, reaching 130 F cm^−3^ and 6 Wh L^−1^ while preserving the advantages of a binder-free architecture [19]. More recently, Peng et al. reported porous lignin-based CNFs delivering 251.5 F g^−1^ at 0.5 A g^−1^ and 5.5 Wh kg^−1^ in a symmetric device, with 95.4% capacitance retention after 10,000 cycles [34]. Al-Majarsh et al. further increased electrode capacitance to 273 F g^−1^ at 1 A g^−1^ through lignin fractionation and mild modification, with a symmetric device reaching 24.6 Wh kg^−1^ [35].

Accordingly, this work presents a simple sustainable strategy for fabricating free-standing, binder-free lignin-derived carbon nanofiber mats with a hierarchical micro–mesoporous architecture through electrospinning of a lignin/PEO precursor, followed by oxidative stabilization, carbonization, and selective acid washing. During thermal treatment, sodium species incorporated through the spinning solution are transformed and subsequently removed by acid washing, promoting pore development without requiring an additional post-treatment carbonization activation step. Rather than maximizing BET surface area alone, this strategy aims to develop an accessible hierarchical pore structure that combines microporous charge-storage domains with mesopores facilitating ion transport. The carbon nanofibers were evaluated as binder-free supercapacitor electrodes in 3 M KOH to establish the relationship between their micro–mesoporous architecture and electrochemical performance. The resulting LCF-AW electrodes reach a specific capacitance of 275 F g^−1^ at 0.25 A g^−1^, together with ~87% capacitance retention at 2.5 A g^−1^ and excellent cycling stability, demonstrating competitive electrochemical performance without conventional post-carbonization chemical activation. Particular attention is given to the balance between microporous surface area and mesopore volume and its influence on capacitance. This study assesses sodium assisted in situ pore formation as a straightforward route to tailor electrochemically accessible porosity without applying a separate conventional activation treatment.

## 2. Materials and Methods

### 2.1. Materials

Kraft lignin (technical grade, Lignin alkali, cat. no. 471003-100G, CAS 8068-05-1), polyethylene oxide (PEO, average Mv 100,000 g mol^−1^, cat. no. 181986-250G, CAS 25322-68-3), sodium hydroxide (NaOH, 99.35%, ACS Reagent, pellets (anhydrous), ≥98%, cat. no. S5881-500G, CAS 1310-73-2), urea (≥98%, cat. no. U5378-500G, CAS 57-13-6, MW: 60.06 g/mol), and hydrochloric acid (HCl, 35 wt% aqueous solution, CAS 7647-01-0) were purchased from Sigma-Aldrich. Conductive carbon black (C-NERGY SUPER C65, cat. no. NG04CO08029) was obtained from Nanografi (Ankara, Turkey). Polytetrafluoroethylene (PTFE, 60 wt.% dispersion in H_2_O, cat. no. 665800-100ML, CAS 9002-84-0) and methanol (MeOH, ≥99.8%, CAS 67-56-1) were also supplied by Sigma-Aldrich (Saint Louis, MO, USA). All chemicals were used as received without further purification.

### 2.2. Preparation of the Electrospun Solution

The spinning solution was prepared in a two-stage process. First, 1.0 g of poly(ethylene oxide) was dissolved in 20 mL of an aqueous NaOH solution (pH = 12) under constant stirring at 30 °C for 1 h. Subsequently, 0.5 g of urea was added, and once fully dissolved, 4.0 g of lignin was gradually incorporated. The mixture was stirred for an additional 2 h at 30 °C until a homogeneous, dark-brown solution was obtained. The total solid concentration was 25 wt.%, with a lignin-to-PEO mass ratio of 80:20 (*w*/*w*), while the urea content was fixed at 10 wt.% relative to the total solid mass (lignin + PEO). These formulation parameters were selected to ensure adequate solubilization of the components and to facilitate the subsequent electrospinning process.

### 2.3. Fabrication of Electrospun Lignin Nanofibers

The as-prepared solution was electrospun using a Spinbox system (Bioinicia, Paterna, Spain). Electrospinning was performed by applying a voltage of 17 kV and a solution flow rate of 1.0 mL h^−1^, controlled using a syringe pump. A stainless-steel needle (14 G, inner diameter of 1.51 mm) was used, and the tip-to-collector distance was fixed at 20 cm. The fibers were collected on a stationary square collector covered with aluminum foil. The temperature and relative humidity inside the electrospinning chamber were 25 °C and 34%, respectively. The resulting fiber mat was dried under vacuum overnight at room temperature and stored in a desiccator prior to further characterization and processing. A schematic representation of the electrospinning setup and the main processing parameters, together with representative images and morphological characterization of the resulting fibers, is provided in Figure S1.

### 2.4. Stabilization and Carbonization

The as-spun nanofibers were carefully peeled from the aluminum foil, cut into rectangular specimens (4 × 9 cm), and placed between carbon felt sheets prior to thermal treatment in a tubular furnace. Stabilization was carried out in air at 200 °C for 2 h using a heating rate of 1 °C min^−1^. The stabilized mats were subsequently carbonized at 800 °C for 2 h under flowing nitrogen (0.7 L min^−1^) at a heating rate of 5 °C min^−1^. After carbonization, the carbon nanofiber mats were washed with distilled water at 85 °C for 1 h to remove water-soluble impurities and residual by-products, rinsed with ethanol, and dried overnight in a vacuum oven at 100 °C.

### 2.5. Acid-Assisted Pore Development

Following the initial water-washing step, a significant fraction of sodium-containing species remained within the carbon nanofibers, either as poorly water-soluble compounds (predominantly sodium carbonate formed during thermal treatment) or trapped within the carbon pore network. To remove these species, the carbon nanofiber mats were immersed in 2 M HCl at room temperature under continuous stirring for 2 h. The acid treatment dissolved the residual sodium compounds (e.g., Na_2_CO_3_ + 2 HCl → 2 NaCl + H_2_O + CO_2_), thereby removing the sodium species responsible for the in situ templating effect during carbonization and promoting the development of the hierarchical pore structure [36]. Finally, the samples were thoroughly washed with distilled water until neutral pH was reached to remove residual acid and soluble by-products (mainly NaCl), and then dried overnight in a vacuum oven at 100 °C. The electrospun lignin fibers, carbonized fibers, and acid-washed carbonized fibers are hereafter denoted as LF, LCF, and LCF-AW, respectively.

### 2.6. Characterization

The morphology and microstructure of both the as-spun and carbonized fibers were characterized using Scanning electron microscopy (SEM, FEI Teneo, Hillsboro, OR, USA), equipped with an energy-dispersive X-ray spectroscopy (EDX) detector. Prior to analysis, all samples were dried overnight under vacuum to remove residual solvent and subsequently sputter-coated with gold (45 s, 15 mA plasma current) to prevent surface charging. SEM micrographs were recorded at an accelerating voltage of 10 kV. Average fiber diameters were calculated using ImageJ^®^ software (version 1.54g, National Institutes of Health, Bethesda, MD, USA) from at least 150 random measurements per sample to ensure statistical significance. The nanostructure of the carbonized materials was further examined by Transmission Electron Microscopy (TEM, Thermo Fisher SPECTRA 200, Hillsboro, OR, USA). The TEM samples were prepared by depositing a dispersion of the carbonized material in ethanol onto holey carbon copper grids.

Fourier transform infrared (FTIR) spectroscopy (FT-IR, JASCO 6X, Tokyo, Japan) was employed to characterize the precursors, as-spun fibers, and carbonized fibers. Spectra were recorded in transmission mode employing the KBr pellet method over the range of 400–4000 cm^−1^, accumulating 128 scans at a spectral resolution of 4 cm^−1^.

Raman spectra and maps were acquired using a confocal Raman microscope (WITec Alpha 300RA, Ulm, Germany). Measurements were performed using a 532 nm excitation laser with a laser power of 0.2 mW focused through a 100× objective lens. Raman maps (5 × 5 µm^2^) were collected with a step size of 0.2 µm, yielding 625 spectra per map and an integration time of 2 s per pixel. Eight regions were analyzed for each sample to ensure statistical representativeness. The spectra were processed using WITec Control Plus software 2.08 by applying cosmic-ray removal (CRR) and background subtraction (BSub) prior to calculating the *I_D_/I_G_* ratio for each Raman map.

The thermal behavior of the precursors and as-spun fibers was analyzed by Differential Scanning Calorimetry (DSC, Mettler Toledo DSC 822, Greifensee, Switzerland) over a temperature range of 25–400 °C at a heating rate of 10 °C min^−1^ under a nitrogen flow, using around 10 mg of sample sealed in aluminum pans. All the DSC curves reported in this work correspond to the first heating scan. The thermal stabilities, mass loss events, oxidative stabilization process, and resulting carbon yield of the fibers were investigated by thermogravimetric analysis (TGA Q50, TA Instruments, New Castle, DE, USA). Samples were heated from 35 to 800 °C at a heating rate of 10 °C min^−1^ under a N_2_ atmosphere. Additionally, to simulate the oxidative stabilization process, the as-spun fibers were heated to 200 °C at 1 °C min^−1^ under an air flow, followed by heating up to 800 °C at 5 °C min^−1^ under a N_2_ atmosphere.

X-ray Photoelectron Spectroscopy (XPS) was performed using a SPECS GmbH spectrometer equipped (Berlin, Germany) with an ultra-high-vacuum (UHV) system and a PHOIBOS 150 9MCD (SPECS GmbH, Berlin, Germany) energy analyzer. A non-monochromatic Mg Kα X-ray source operated at 200 W and 12 kV was used. High-resolution spectra were deconvoluted using KherveFitting (v1.8) [37] after Shirley background subtraction. The binding energy scale was calibrated against the C 1s C–C/C=C peak at 284.8 eV. The textural properties of the carbon nanofiber mats were determined from N_2_ adsorption–desorption isotherms measured at 77 K on a Micromeritics ASAP 2020 instrument (Norcross, GA, USA). Prior to analysis, the samples were degassed under vacuum at 150 °C for at least 4 h. Specific surface area was calculated by the Brunauer−Emmett−Teller (BET) method, whereas pore size distribution was derived using Nonlocal Density Functional Theory (NLDFT).

### 2.7. Electrochemical Testing

The electrochemical performance of the self-standing electrodes was evaluated in a Swagelok T-type cell using a Bio-Logic SP-150e potentiostat (Biologic Scienc Instruments, Seyssinet-Pariset, France), employing two complementary configurations. A three-electrode configuration was used for cyclic voltammetry (CV), galvanostatic charge–discharge (GCD), and electrochemical impedance spectroscopy (EIS), which allowed the single-electrode response to be resolved against a reference electrode; a symmetric two-electrode configuration was then used to assess the long-term cycling stability of the assembled device. In both cases, two nominally identical discs of the acid-treated lignin carbon fibers (cut with a 3 mm hole punch, with closely matched active masses of 0.00023 ± 0.0001 g per electrode) were used, two glassy carbon disks (BASi) served as current collectors, and a Whatman separator soaked in 3 M KOH was placed between them. In the three-electrode configuration, the two discs acted as working and counter electrodes, and an Ag/AgCl (3 M KCl) electrode placed at the T-junction served as the reference.

In the three-electrode configuration, cyclic voltammetry was first used to establish the operating potential window of the material, which was set to −1.0 to 0.0 V vs. Ag/AgCl 3 M KCl. The electrodes were conditioned by 30 CV cycles to activate the material, after which CV curves were recorded at different scan rates (5, 10, 25, 50, 100, 150, and 200 mV s^−1^) to evaluate the electrochemical response as a function of scan rate. The specific capacitance was also derived from the CV curves by the area-integration method, as described in Equation (1), [38]:

(1) $$C_{s}\left(F\;g^{-1}\right)=\frac{\int I\;dV}{2\cdot m\cdot \upsilon \cdot ∆V}$$

where ∫I dV is the area under the CV curve, *m* is the electrode mass of a single electrode, *υ* is the scan rate, and ∆*V* is the operating potential window. The factor of 2 accounts for the complete CV cycle (charging and discharging branches).

Galvanostatic charge–discharge (GCD) measurements, also referred to as chronopotentiometry (CP), were subsequently carried out at different current densities (0.25, 0.5, 1.0, 1.5, 2.0 and 2.5 A g^−1^) in the −1.0 to 0.0 V vs. Ag/AgCl 3 M KCl range, to determine the specific capacitance from Equation (2), [39]:

(2) $$C_{s}\left(F\;g^{-1}\right)=\frac{I\cdot t}{m\cdot ∆V}$$

where *I* is the applied current, *t* is the discharge time (taken after IR-drop correction), *m* is the active mass of a single electrode, and Δ*V* is the operating potential window (excluding the IR drop).

From the charge–discharge curves, the energy and power densities were quantified for their representation in a Ragone plot. These figures of merit were derived from the galvanostatic profiles measured at the various current densities, with the gravimetric energy density calculated according to Equation (3), [40]:

(3) $$E\;\left(Wh\;{kg}^{-1}\right)=\frac{1}{2}\cdot C_{s}\cdot {∆V}_{cell}^{2}\cdot \frac{1000}{3600}$$

where *C_s_* is the specific capacitance obtained from the charge–discharge curves at each current density, and Δ*V_cell_* is the cell voltage window. For device-level evaluation, a symmetric two-electrode configuration was assumed, with both electrodes operating over opposite potential ranges; the total cell voltage window was therefore taken as twice the single-electrode window. The gravimetric power density was then obtained from Equation (4), [40]:

(4) $$P\;\left(W\;{kg}^{-1}\right)=E\cdot \frac{3600}{∆t}$$

Electrochemical impedance spectroscopy (EIS) measurements were performed after electrode conditioning by applying a sinusoidal perturbation of 5 mV over the frequency range from 200 kHz to 100 mHz, with six points per decade. The spectra were recorded at the open-circuit potential (OCP).

Finally, the cycling stability was evaluated in a symmetric two-electrode configuration over 10,000 galvanostatic charge–discharge cycles at a current density of 10 A g^−1^, monitoring both the coulombic efficiency and the capacitance retention.

## 3. Results and Discussion

### 3.1. Morphology, Structural, and Textural Properties of the Lignin Fibers

**Figure 1:**
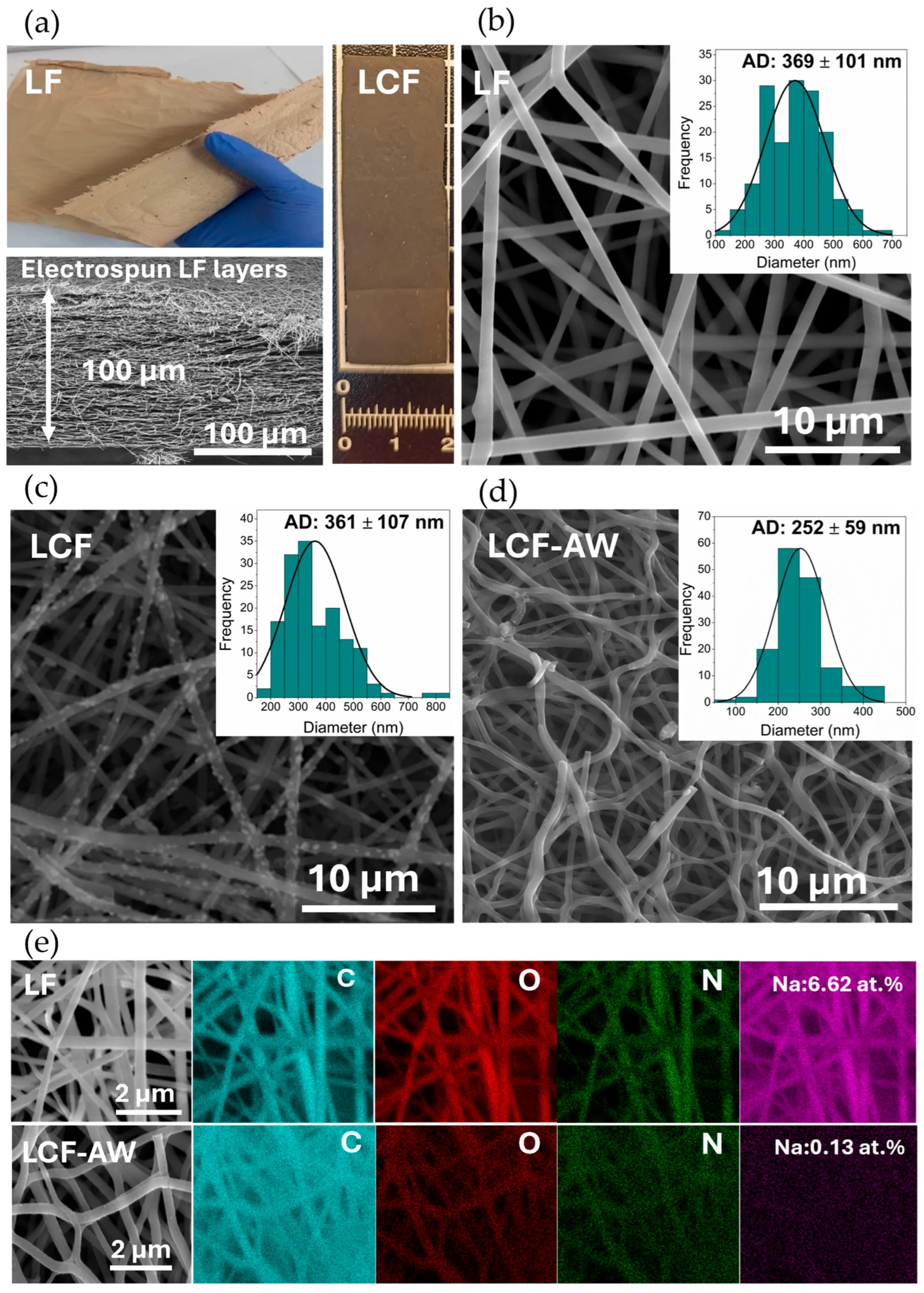
(**a**) Digital photograph of the as-spun lignin fiber (LF) and lignin carbon fiber after furnace treatment (LCF) mats and a representative cross-sectional SEM image of the LF precursor. SEM micrographs of (**b**) LF, (**c**) carbonized fiber before washing (LCF), and (**d**) after washing (LCF-AW). Insets in (**b**–**d**) display the corresponding fiber diameter distribution histograms. (**e**) EDX elemental mapping of C, O, N, and Na: LF (up) and LCF-AW (down).

Lignin-based nanofibers were successfully fabricated via electrospinning a 25 wt.% lignin/PEO solution (80:20 *w*/*w*) in a 0.5 M aqueous NaOH solvent system. NaOH was incorporated into the lignin/PEO spinning solution to provide sodium species that were retained and evolved upon thermal treatment, contributing to in situ pore formation after the subsequent acid-washing step. The use of an aqueous NaOH solvent has been demonstrated to yield continuous, bead-free lignin fibers suitable for subsequent carbonization [32]. Figure 1 shows the as-spun nanofiber mat together with the corresponding carbonized sample. The precursor mat was approximately 100 µm thick (Figure 1a) and consisted of multiple stacked fibrous layers composed of homogeneous, bead-free fibers with an average diameter of 369 ± 101 nm (Figure 1b).

SEM images of the carbonized fibers (Figure 1c) reveal numerous granular surface deposits that increase the apparent surface roughness while preserving the overall fibrous morphology. These features are consistent with the formation of sodium-containing phases during carbonization [19]. The average fiber diameter decreased only slightly to 361 ± 107 nm despite the substantial mass loss associated with the thermal conversion of the lignin/PEO precursor. The relatively limited fiber shrinkage is attributed to the transient presence of these phases, which partially offset the contraction of the carbon framework [14,41]. Similar surface features have recently been reported for electrospun organosolv lignin/PEO fibers prepared from alkaline solutions and identified by EDX and XRD as sodium-rich crystalline domains, predominantly composed of Na_2_CO_3_ [42].

Subsequent HCl washing (LCF-AW) preserved the overall fibrous morphology while reducing the average fiber diameter to 252 ± 59 nm (Figure 1d), corresponding to approximately a 30% decrease relative to the unwashed LCF. This diameter reduction, along with the narrower size distribution (from 107 to 59 nm), is attributed to selective dissolution of the sodium-containing phases, previously embedded within or deposited on the fibers [36,43]. Despite this contraction, the fibers remained continuous and free of significant structural damage, indicating that acid leaching efficiently removed the inorganic phases without compromising the integrity of the carbon framework. Although the resulting porosity cannot be directly resolved by SEM at this magnification, removing these sodium-containing phases is expected to generate the hierarchical pore structure later confirmed by the textural analysis [36,44].

The dispersion of Na-containing species within the precursor fibers provides an extended interface with the lignin-based matrix during carbonization. During thermal treatment, these species may undergo chemical and structural transformations, including the formation of Na-containing [19,42]. Their subsequent removal by HCl washing contributes to the development of accessible porosity, as supported by the marked increase in surface area and pore volume after washing. Consistently, the XRD pattern of LCF-AW shows no detectable crystalline reflections attributable to Na_2_CO_3_ (Figure S2), in agreement with the very low residual Na content determined by EDX and XPS. Therefore, the pronounced increase in microporosity is more appropriately attributed to sodium-assisted pore development, potentially involving both the formation and subsequent removal of Na-containing phases and chemical interactions between NaOH-derived species and the carbon framework.

We also investigated the elemental composition and spatial distribution of the fibers by EDX elemental mapping, comparing the as-spun precursor (LF) with the acid-washed carbonized fibers (LCF-AW) (Figure 1e and Table S1). Both samples exhibited a homogeneous distribution of carbon and oxygen throughout the fibrous network. After carbonization, the lower relative oxygen signal is consistent with the progressive deoxygenation and aromatization of the lignin-derived carbon framework. Nitrogen was not quantitatively evaluated by EDX because the low-energy N Kα emission and the limited sensitivity of EDX for light elements in a carbon-rich matrix result in considerable uncertainty. Therefore, nitrogen content was assessed by XPS, as discussed below. Most importantly, EDX provided direct evidence for the effectiveness of the acid-leaching treatment. The sodium content, originating from the alkaline spinning solution, decreased from 6.62 at.% in the electrospun fibers to only 0.13 at.% after carbonization and HCl washing, as evidenced by the almost complete disappearance of the Na signal in the elemental maps. This marked depletion supports the proposed role of sodium-containing phases as transient in situ templates that are selectively removed after thermal treatment and subsequent acid-washing, promoting the development of the hierarchical micro–mesoporous structure of the carbon nanofiber mats.

**Figure 2:**
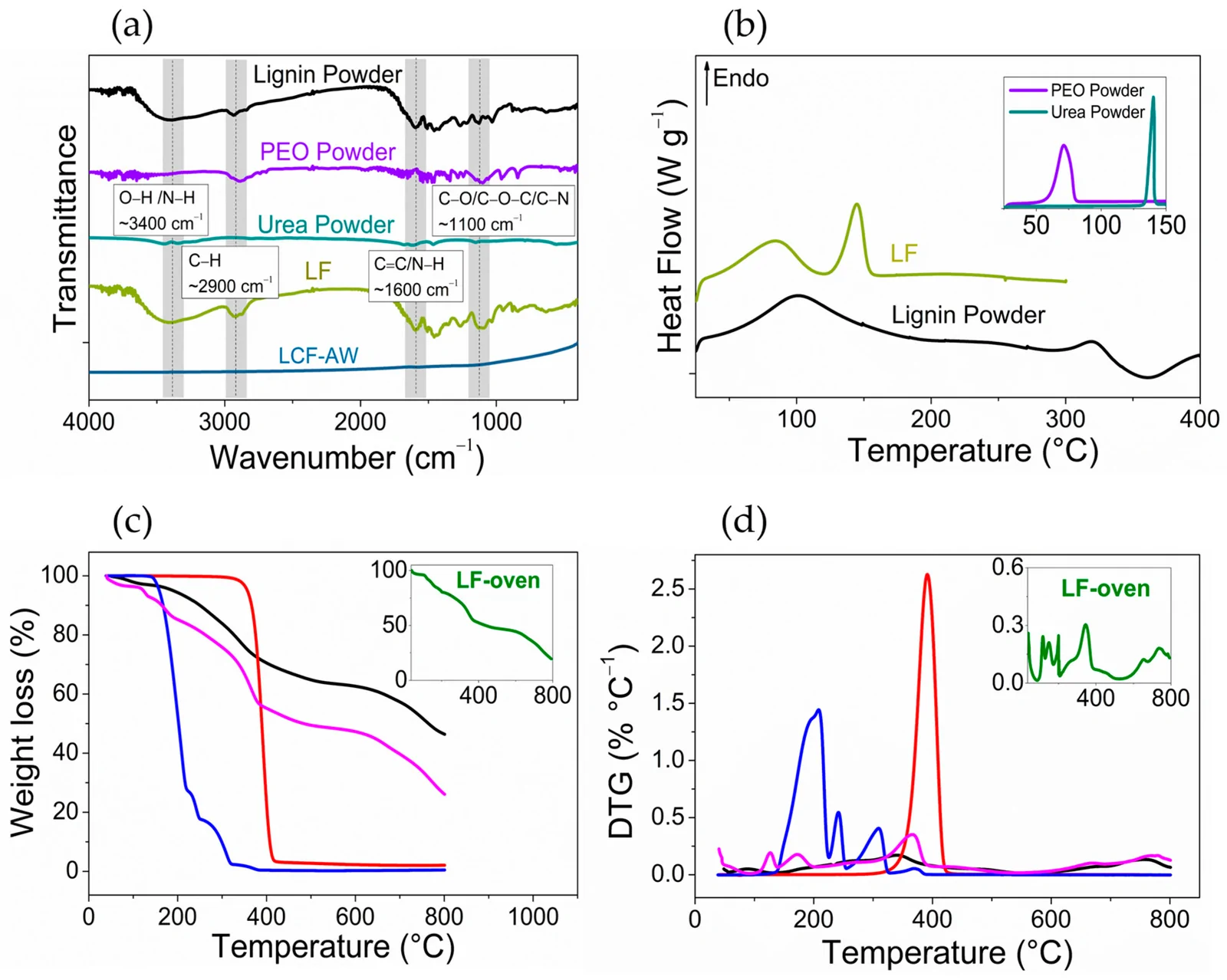
(**a**) FTIR spectra of lignin, PEO, urea, lignin fiber (LF), and carbonized fiber after washing (LCF-AW). Grey-shaded areas indicate the main absorption regions and their corresponding assignments. (**b**) DSC curves of lignin, PEO, urea, and LF. (**c**) TGA and (**d**) DTG curves of lignin (–), PEO (–), urea (–), and LF (–) under N_2_. Inset shows the LF-oven (air to 200 °C followed by N_2_).

FTIR spectroscopy was used to monitor the chemical evolution of the precursor components, the electrospun fibers, and the resulting carbon nanofibers during thermal conversion (Figure 2a and Table S2). The spectrum of lignin displayed the characteristic bands of lignocellulosic materials, including the broad O–H stretching vibration centered at 3400 cm^−1^, aliphatic C–H stretching at 2900 cm^−1^, aromatic C=C stretching vibrations around 1600 cm^−1^ [45], and C–O vibrations associated with phenolic and alcoholic groups in the 1200–1000 cm^−1^ region [45,46]. PEO exhibited C–H stretching vibrations at 2880 cm^−1^, an intense ether C–O–C stretching band at 1100 cm^−1^ (appearing as a triplet-shaped peak), and crystalline CH_2_ rocking vibrations at 955 and 842 cm^−1^ [47]. The urea spectrum showed the characteristic N–H stretching bands (3440–3340 cm^−1^), N–H bending vibration at 1606 cm^−1^, and C–N stretching bands at 1457 and 1146 cm^−1^ [48,49], confirming its identity in the precursor formulation.

The FTIR spectrum of the electrospun precursor fibers (LF) largely retains the characteristic absorption regions of lignin, PEO, and urea, with only minor differences compared with the individual components. This limited spectral variation is mainly attributed to the strong overlap of their characteristic bands, particularly in the O–H/N–H (~3400 cm^−1^), C–H (~2900 cm^−1^), and C–O/C–O–C/C–N (~1100 cm^−1^) regions (Figure 2a). Nevertheless, some broadening of the O–H/N–H and C–O/C–N absorption regions is observed in LF, consistent with intermolecular interactions among the components in the electrospun fibers. Thus, spectral similarity is expected for this multicomponent system and does not indicate the absence of interactions between its constituents.

After carbonization and acid washing (LCF-AW), the characteristic bands of PEO and urea disappeared almost completely, while the oxygen-containing functional groups of lignin were markedly reduced, reflecting the thermal decomposition of sacrificial components and the aromatization of the lignin framework into a carbonaceous structure [47,50]. The characteristic PEO and urea bands are no longer detectable after carbonization, consistent with the extensive removal of these components during thermal treatment.

The thermal behavior of the individual precursors and the lignin fiber (LF) was examined by differential scanning calorimetry (DSC) (Figure 2b and Table S3). All samples were vacuum-dried prior to DSC analysis. The reference thermogram of PEO exhibited the characteristic melting endotherms expected for semicrystalline polymers centered at ~70 °C, consistent with the literature values [51]. Urea displayed a sharp endotherm peak centered at ~140 °C, corresponding to the melting of its crystalline phase and in good agreement with the reported melting temperature (133 °C) [52].

For both kraft lignin and the electrospun lignin fibers (LF), only the first heating scan was analyzed, as subsequent heating cycles induce irreversible structural changes that alter the original fibrous morphology and thermal state of the material. Lignin displayed a broad endothermic event starting around 90 °C, attributed to the glass transition (typically reported at 90–170 °C depending on its origin and composition), together with the release of physically bound residual moisture [50]. After the glass transition, the increased molecular mobility facilitates condensation and cross-linking reactions, which become progressively more pronounced above approximately 200 °C. Above ~300 °C, these reactions proceed simultaneously with thermal decomposition, leading to the formation of a carbonaceous structure [53,54].

The DSC thermogram of the electrospun lignin fibers (LF) combined the characteristic thermal events of its individual constituents. In addition to the thermal transition associated with lignin, a distinct endothermic peak appeared at approximately 144 °C, close to the melting transitions of both PEO and urea. The persistence of this feature indicated that both additives remained as discrete domains within the as-spun fiber rather than being completely molecularly dispersed throughout the lignin matrix. Their successful incorporation is consistent with the FTIR results and indicates that both additives remain available for thermal decomposition during carbonization.

The thermal stability and decomposition behavior of the individual precursors and the LF were investigated by thermogravimetric analysis (TGA) under a nitrogen atmosphere up to 800 °C (Figure 2c,d and Table S4). For comparison, the LF-oven sample corresponds to the same electrospun precursor heated under air up to 200 °C and subsequently under nitrogen, allowing evaluation of the influence of the initial oxidative treatment on the subsequent carbonization process. The individual precursors provide the basis for interpreting the thermal evolution of the multicomponent fiber. Urea underwent a multistep decomposition between approximately 190 and 370 °C under nitrogen, as reflected by the DTG peaks at 204, 243, 307, and 370 °C (Figure 2d) [55]. In contrast, PEO decomposed rapidly in a single stage between 300 and 440 °C, leaving negligible residue (~2 wt.%). The DTG peak centered at approximately 392 °C was consistent with the random chain rupture well above its melting point (65 °C) [56]. In contrast, kraft lignin showed a much broader degradation profile and retained about 46 wt.% residue at 800 °C, reflecting its heterogeneous aromatic structure and progressive carbonization [57].

The electrospun fiber (LF) displayed a multistep degradation profile arising from the combined thermal evolution of its constituents. After a minor mass loss below 120 °C, mainly associated with residual moisture, degradation proceeded gradually between 150 and 300 °C owing to urea decomposition together with the onset of lignin condensation [55,57]. The main degradation stage occurred between 350 and 500 °C, where the DTG reached its maximum at 366 °C. This region corresponded to the overlap between PEO decomposition and the principal pyrolysis of lignin, leading to the removal of the sacrificial additives while the lignin matrix progressively carbonized. Consequently, the decomposition of PEO and urea may generate transient free volume through the release of volatile species during carbonization; however, their specific contribution to the final pore architecture cannot be independently quantified from the present experimental design. Meanwhile, lignin undergoes progressive carbonization, preserving the fibrous framework and yielding a carbon residue of approximately 26 wt.% at 800 °C [57,58].

The LF-oven sample exhibited a TGA profile similar to that of LF under nitrogen, with a slightly earlier onset of degradation, but a lower final residue (~20 wt.% versus 26 wt.%), indicating limited thermo-oxidation during the initial heating stage in air without significantly altering the subsequent carbonization pathway [51].

**Figure 3:**
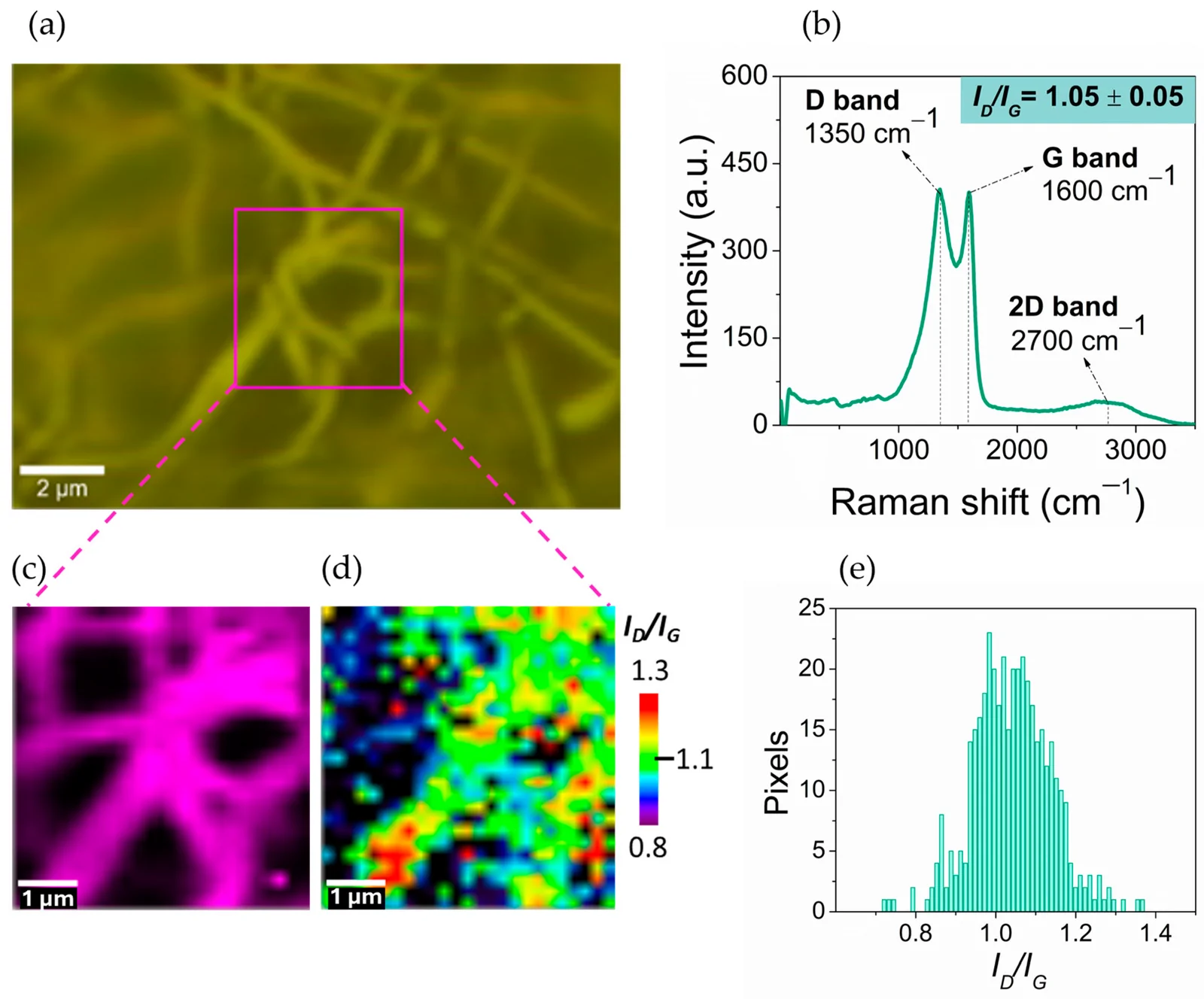
(**a**) Optical image acquired by confocal Raman microscopy of LCF-AW showing the region selected for Raman mapping; (**b**) Average Raman spectrum of the mapped region; (**c**) Raman phase map of the analyzed area; (**d**) Spatial distribution of the *I_D_*/*I_G_* ratio obtained from Lorentzian fitting of the Raman spectra; and (**e**) Histogram showing the statistical distribution of the *I_D_*/*I_G_* ratio.

Figure 3 presents the confocal Raman microscopy analysis of the acid-washed lignin-derived carbon fibers (LCF-AW), where the highlighted square in the optical image (Figure 3a) indicates the region selected for Raman mapping. The average Raman spectrum of the highlighted region (Figure 3b) clearly displayed the three characteristic carbon bands: the defect-induced D band (~1350 cm^−1^), the graphitic G band (~1600 cm^−1^), and the 2D band (~2700 cm^−1^) commonly associated with graphitic ordering and layer stacking [59]. The mean *I_D_*/*I_G_* ratio (1.05 ± 0.05) was obtained from approximately 5000 individual Raman spectra collected over eight independent maps, indicating excellent experimental reproducibility and a high degree of structural homogeneity.

The *I_D_*/*I_G_* ratio is characteristic of a significant degree of structural disorder and a high density of defects and edge sites, which is characteristic of lignin-derived porous carbons and consistent with their highly porous and disordered carbon framework [60]. This is consistent with the presence of small sp^2^ domains with a large proportion of edge carbon atoms. In contrast, the weak and poorly defined 2D band confirmed a low degree of graphitization and an absence of well-ordered graphitic stacking [61]. Such a defect-rich structure is generally considered beneficial for electrochemical applications because it provides abundant electrochemically active sites that facilitate charge storage [62].

The Raman phase map based on the D-band intensity (Figure 3c) clearly reproduced the morphology observed in the optical image. Furthermore, the *I_D_*/*I_G_* spatial map derived from Lorentzian fitting (Figure 3d) revealed a largely uniform disorder distribution, with most values clustered around 1.1, indicating that most pixels were clustered *I_D_*/*I_G_* ≈ 1.05, with only a limited number of higher and lower values. This high spatial uniformity confirms the microstructural homogeneity of the carbon nanofiber mat, which is expected to promote a more homogeneous distribution of electrochemically active sites throughout the electrode.

**Figure 4:**
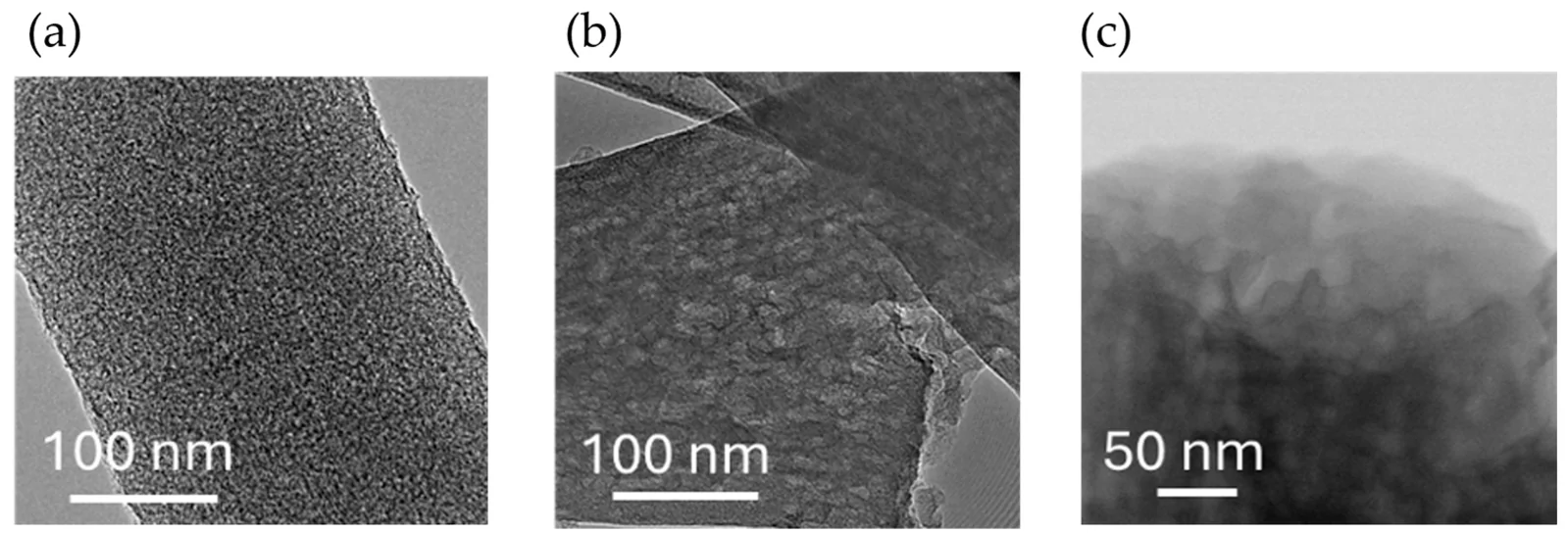
HR-TEM images of the carbonized fibers (**a**) before acid washing (LCF); (**b**) after acid washing (LCF-AW); and (**c**) cross-sectional image of an acid-washed fiber showing the internal porous structure.

High-resolution transmission electron microscopy (HR-TEM) was used to visualize the morphological changes induced by acid leaching, and to assess the development of porosity within the carbon fibers (Figure 4). Prior to the acid treatment (Figure 4a), the carbonized fibers (LCF) exhibited a predominantly dense internal matrix with restricted visible porosity. This morphology is consistent with sodium-containing phases formed during carbonization, in agreement with the SEM and EDX results discussed above. Such structural behavior has been widely reported in templated biomass-derived carbons, where inorganic species act as transient, space-filling structure-directing agents before chemical and thermal treatment [11,14].

Following HCl washing (Figure 4b), a pronounced change in the internal morphology became evident. The TEM image of LCF-AW revealed a well-developed porous network within the fiber, with an apparently interconnected morphology. This morphology is consistent with the selective removal of the sodium-containing phases during acid washing. Similar template-assisted pore formation has been widely reported [14,63].

**Figure 5:**
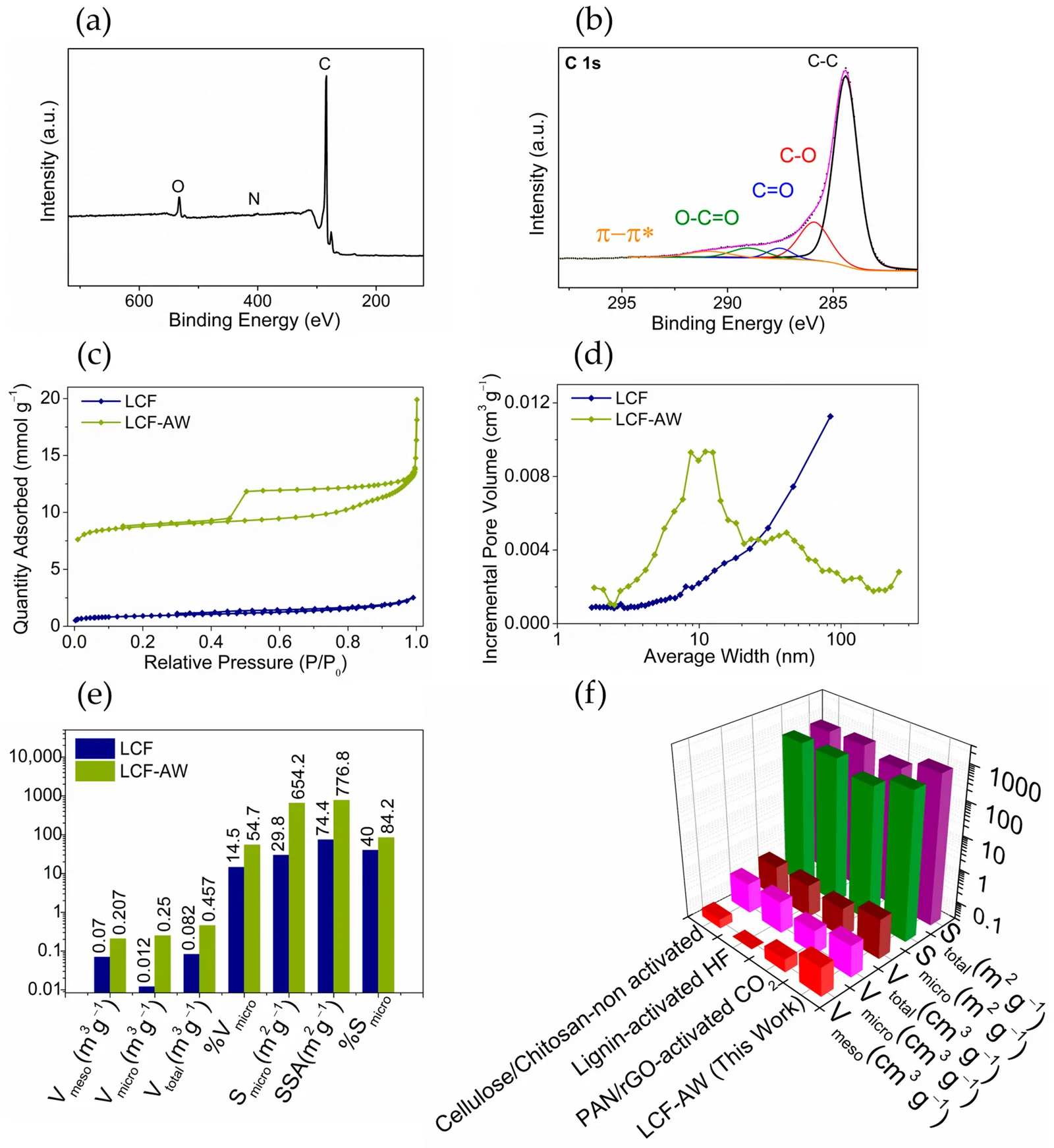
(**a**) XPS survey spectrum of LCF-AW; (**b**) high-resolution C 1s spectrum with peak deconvolution; (**c**) N_2_ adsorption–desorption isotherms at 77 K; (**d**) NLDFT pore-size distributions; (**e**) comparison of textural parameters (surface area and pore volumes) before (LCF) and after acid washing (LCF-AW); and (**f**) comparison of the textural properties of LCF-AW with representative electrospun carbon fibers reported in the literature, including PAN/rGO active CO_2_ [75], lignin active HF [24], and cellulose/chitosan [76].

Higher-magnification cross-sectional TEM analysis (Figure 4c) further revealed the porous internal structure of the acid-washed fibers. Although TEM cannot directly resolve micropores because of their small size, the observed contrast variations indicate that pore development extends throughout the fiber interior rather than being restricted to the external surface [12,14]. This observation is consistent with the development of a micro- and mesoporous network whose pore-size distribution was quantitatively assessed by N_2_ adsorption analysis (Figure 5c,d, discussed below).

In addition to sodium-assisted templating, PEO decomposition may generate transient free volume within the fibers during carbonization, although its specific contribution to the final pore architecture cannot be independently established. In contrast, the pronounced development of accessible porosity after acid washing may be associated with the removal of sodium-containing phases. Similar synergistic effects between sacrificial polymers and inorganic templates have been widely reported for lignin-derived porous carbon fibers, as well as the role of sacrificial block-copolymer-derived carbon fibers [64,65].

X-ray photoelectron spectroscopy (XPS) analysis provides evidence of the surface chemical composition of the carbonized fibers. The survey spectrum (Figure 5a) confirmed the presence of three peaks corresponding to C 1s, N 1s, and O 1s at 284.3, 401.1, and 533.1 eV, respectively. The surface atomic composition derived from this survey consisted of 94.7 at% C, 4.2 at% O, and only 1.0 at% N, indicating an extensive carbonization process and a limited concentration of surface heteroatoms. The N 1s signal was barely distinguishable from the baseline, identifying nitrogen as a minor surface constituent of the carbonized fibers.

The C 1s envelope (Figure 5b) was deconvoluted into five distinct components. The dominant peak at 284.4 eV (69.5% of the total C 1s area) was assigned to sp^2^/sp^3^ carbon in aromatic and aliphatic C–C and C=C environments, representing the condensed carbon network formed during lignin carbonization [66,67]. The component at 285.9 eV (17.7%) corresponded to carbon singly bonded to oxygen or nitrogen (C-O in phenolic, alcoholic and ether groups, and C–N), in line with the C–O/C–N positions reported for carbons and carbon fibers. Minor contributions at 287.5 and 289.0 eV (~12% in total) were assigned to carbonyl (C=O) and carboxyl/ester (O–C=O/COOH) groups, respectively. The prevalence of the C–C/C=C contribution and the small intensity of the oxygenated components served as a diagnostic indicator of the extensive deoxygenation and the growth of the aromatic framework that accompany carbonization of lignocellulosic precursors [68,69]. A weak π–π* shake-up satellite centered at approximately 290.8 eV (5.2%) also supports the presence of extended aromatic conjugation, in agreement with the partial graphitization of the carbon nanofibers.

The O 1s envelope (Figure S3a) was deconvoluted into four components assigned to doubly bonded oxygen in carbonyl-, quinone-, ketone-, and lactone-like structures (531.2 eV), singly bonded oxygen in phenolic, hydroxyl, and ether groups (532.4 eV), and highly oxygenated surface functionalities, associated with O–C–O, ester, and carboxyl environments (533.6 eV), together with a minor high-binding-energy contribution at 535.7 eV (<10.0%) attributed to adsorbed water. Such assignments are consistent with previous XPS studies on carbonaceous materials [70,71]. Owing to the overlap of several oxygen-containing functionalities in the 532–534 eV region, these assignments should be regarded as indicative. The O 1s results indicate that the residual surface oxygen is present mainly as phenolic and ether functionalities, with a smaller contribution from more highly oxidized groups. Together with the low oxygen content determined from the survey spectrum, this suggests that the remaining oxygen-containing groups are preferentially located at defect sites and exposed edges generated during carbonization, rather than being homogeneously distributed throughout the carbon framework. These observations are consistent with the progressive deoxygenation and aromatization of lignin during thermal treatment [68,69].

The N 1s region (Figure S3b) exhibits a weak and broad signal centered at approximately 400.3 eV. Because of the low nitrogen concentration and the limited signal-to-noise ratio, reliable deconvolution into individual nitrogen configurations is not justified. Therefore, the spectrum was fitted using a single component representing the overall contribution of residual nitrogen species (pyrrolic, pyridinic, graphitic, and/or quaternary) [69]. The low surface nitrogen content indicates that only a limited fraction of the nitrogen introduced as urea was retained after carbonization. Given its low content, this residual surface nitrogen is unlikely to be the primary factor expected to govern the textural or electrochemical behavior of the material, which is instead expected to be strongly influenced by the engineered pore architecture.

To further verify the pore structure generated, N_2_ adsorption/desorption measurements were performed at 77 K. The isotherms of the carbonized lignin fibers before (LCF) and after acid washing (LCF-AW) are presented in Figure 5c. The untreated LCF exhibited very limited nitrogen uptake across the entire pressure range, indicating the presence of poorly accessible porosity. In contrast, LCF-AW displayed a typical Type IV isotherm with a well-defined hysteresis loop, characteristic of mesoporous materials according to the IUPAC classification [72]. The pronounced adsorption at low relative pressure (P/P_0_ < 0.1) corresponds to micropore filling, whereas the hysteresis loop at intermediate-to-high pressures reflects capillary condensation within mesopores [72]. The abrupt closure of the desorption branch around P/P_0_ ≈ 0.5 is attributed to the cavitation (tensile strength) effect; therefore, only the adsorption branch was considered for the NLDFT pore-size analysis. These results demonstrate that acid washing efficiently removes the sodium-containing species that block the porous network, exposing a well-developed hierarchical pore structure in agreement with the SEM, TEM, and EDX observations discussed above.

Quantitative textural analysis (Figure 5d,e and Table S5) further confirms the effect of the acid-leaching treatment on the pore structure. The BET specific surface area increased by more than one order of magnitude, from 74.4 to 776.8 m^2^ g^−1^, while the total pore volume increased from 0.082 to 0.457 cm^3^ g^−1^. This improvement originated primarily from the development of the microporous network, whose volume increased from 0.012 to 0.250 cm^3^ g^−1^. Consequently, micropores account for approximately 84% of the specific surface area (654.2 m^2^ g^−1^), demonstrating that the remarkable increase in BET surface area is mainly associated with the generation of accessible microporosity [32]. In contrast, the total pore volume is much more evenly distributed, with micropores contributing 55% of *V_total_* (0.250 cm^3^ g^−1^) and mesopores 45% (0.207 cm^3^ g^−1^), indicating that inorganic residues were preferentially lodged within the narrowest pores. The pore-size distribution further reveals a well-defined mesopore population centered between 8 and 12 nm, indicating that the acid treatment not only exposes previously inaccessible micropores but also preserves a substantial mesoporous network. This hierarchical architecture is consistent with the combined effect of the sodium-assisted in situ templating process, the subsequent removal of sodium-containing phases during acid washing, and the intrinsic fibrous morphology generated by electrospinning [32].

This balanced micro–mesoporous structure is particularly advantageous for electrochemical applications. While the micropores provide the large specific surface area required for charge storage, the 8–12 nm mesopores are expected to facilitate electrolyte penetration and shorten ion diffusion pathways, thereby improving the accessibility of the electrochemically active surface [24,73]. Unlike many highly activated carbons, where ultramicropores overwhelmingly dominate the pore structure [74], the present material combines a predominantly microporous surface with a nearly balanced micro/mesopore volume distribution, which is expected to minimize diffusion limitations during charge–discharge processes.

Finally, Figure 5f compares the textural properties of LCF-AW with representative electrospun carbon fibers reported in the literature. Despite the absence of a conventional chemical activation treatment, LCF-AW achieved a BET surface area (776.8 m^2^ g^−1^) comparable to or higher than several activated carbon nanofiber systems, including CO_2_-activated PAN/rGO (373 m^2^ g^−1^) [75], HF-treated lignin fibers (612 m^2^ g^−1^) [24] and non-activated cellulose/chitosan fibers (584.9 m^2^ g^−1^) [76]. More importantly, LCF-AW exhibits one of the highest mesopore volumes (0.207 cm^3^ g^−1^) among the compared materials, exceeding that of the HF-treated lignin systems (0.023 cm^3^ g^−1^, ~93% microporous), which is almost exclusively microporous, and that of the cellulose/chitosan-derived fibers (0.053 cm^3^ g^−1^). This demonstrates that the proposed sodium-assisted strategy not only maximizes the BET surface area but also generates a highly accessible hierarchical pore network in which a substantial mesoporous volume complements the large microporous surface. Such a balance between porosity and pore accessibility is particularly attractive for supercapacitor electrodes. Thus, the proposed sodium-assisted strategy provides a simple and cost-effective route to engineer hierarchical porosity in lignin-derived carbon nanofibers without conventional activation treatments.

### 3.2. Electrochemical Performance of the Lignin Carbonized Fiber

**Figure 6:**
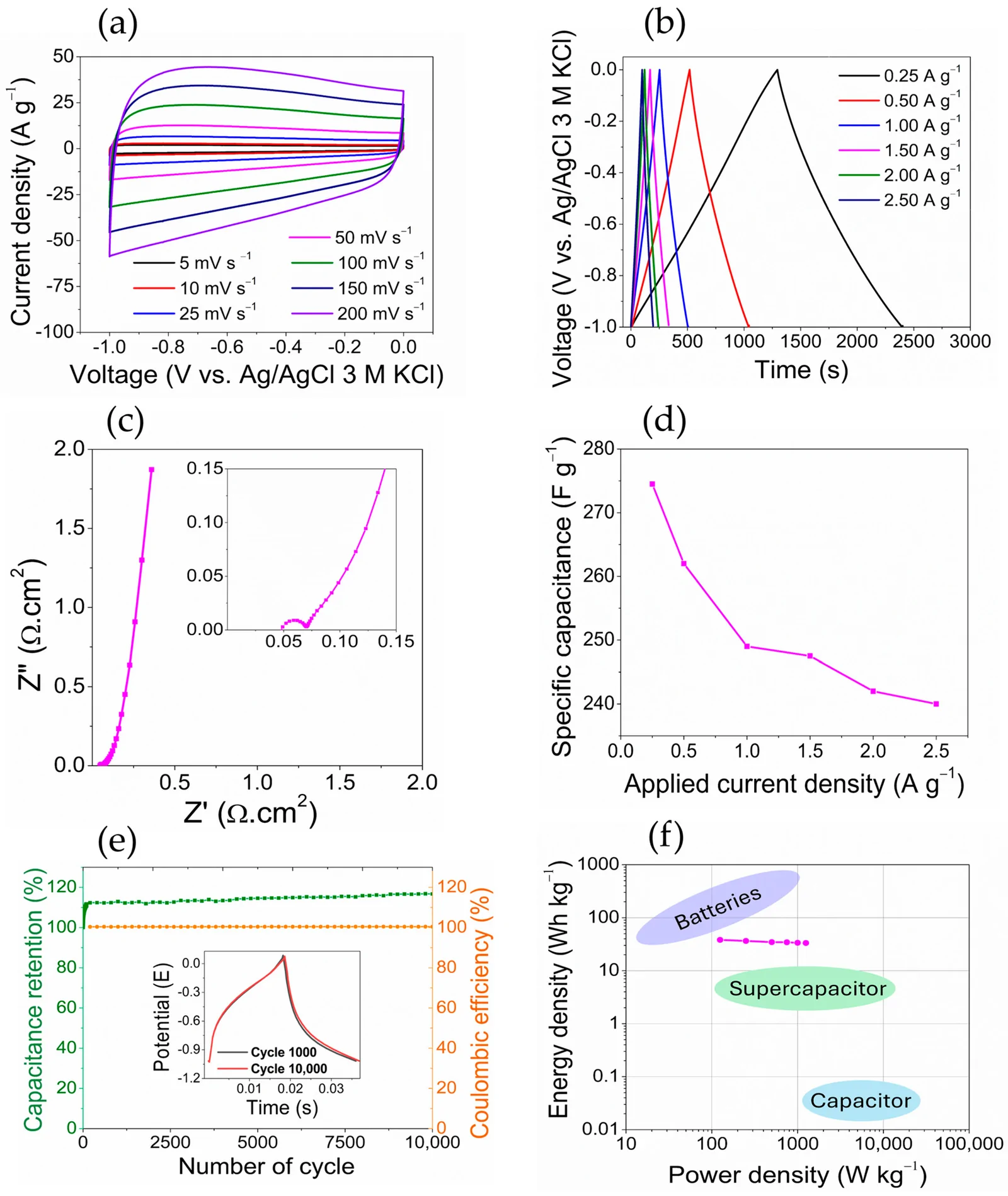
Electrochemical performance of lignin carbonized fiber after HCl washing (LCF-AW). (**a**) CV curves at different sweep rates; (**b**) GCD curves at various current densities; (**c**) Nyquist plot of the PEIS spectrum; (**d**) specific capacitance; (**e**) capacitance retention and coulombic efficiency from electrode stability along 10,000 cycles at a current density of 10 A g^−1^; and (**f**) Ragone plot calculated from GCD traces.

The electrochemical performance of the NaOH-templated lignin-derived carbon nanofibers (LCF-AW) was evaluated in a symmetric three-electrode Swagelok T-type cell using 3 M KOH as the electrolyte (Figure 6). The combination of hierarchical porosity and a conductive carbon framework provides a favorable architecture for electrolyte transport and electric double-layer charge storage, as examined below [13,62].

Aqueous 3 M KOH was selected as the electrolyte because of its high ionic conductivity and mobility of K^+^ and OH^−^ ions, which ensures low internal resistance and efficient ion transport within the porous electrode. In addition, the relatively small hydrated radius of K^+^ facilitates ion access to the micro- and mesopores where the electric double layer forms [77]. A concentration of 3 M was selected because it provides high-conductivity while offering lower viscosity and reduced corrosivity than the more concentrated solutions (6 M) commonly reported [78]. The cyclic voltammograms recorded at scan rates ranging from 5 to 200 mV s^−1^ (Figure 6a) displayed quasi-rectangular profiles without pronounced redox peaks, indicating that charge storage is dominated by electric double-layer capacitance [5,20]. As the scan rate increased, the current response increased proportionally (±40 A g^−1^ across most of the potential window and reaching up to ±60 A g^−1^ near the potential limits). At the same time, the CV curves largely retained their shape, although a slight distortion became apparent at the highest scan rates. This behavior is attributed to the increasing influence of ohmic resistance and ion-diffusion limitations within the microporous network as the charging time decreases, a characteristic feature of micropore-dominated carbon electrodes [13,73]. Despite this, the nearly rectangular response preserved even at 200 mV s^−1^ suggests that the hierarchical pore architecture provides efficient electrolyte access and rapid ion transport throughout the electrode.

The galvanostatic charge–discharge (GCD) curves recorded at current densities between 0.25 and 2.5 A g^−1^ (Figure 6b) exhibited nearly symmetric triangular profiles over the entire potential window, confirming the predominantly capacitive behavior already observed by cyclic voltammetry. As expected, the discharge time decreased progressively from 1300 s at 0.25 A g^−1^ to about 100 s at 2.5 A g^−1^ as the current density increased. Despite the shorter discharge times, the IR drop at the beginning of discharge remained remarkably low (2.6 mV at 2.5 A g^−1^, determined by magnifying the discharge onset), indicating low internal resistance and efficient charge transport within the electrode.

Electrochemical impedance spectroscopy (Figure 6c) provides further insight into the charge-transport properties of the electrode. The Nyquist plot exhibits a very small high-frequency intercept (Rs ≈ 0.05 Ω cm^2^), together with a narrow charge-transfer semicircle (R_ct_ < 0.1 Ω cm^2^), indicating low ohmic and interfacial resistances. In the intermediate-frequency region, a short Warburg-type segment (with a slope approaching 45°) reflects the finite ion-diffusion resistance associated with electrolyte transport through the porous network. At lower frequencies, the nearly vertical line is characteristic of an ideal capacitive response, demonstrating efficient electrolyte penetration throughout the hierarchical porous structure [5,62]. These low resistance values can be attributed to the self-supported binder-free architecture of the carbon nanofiber mat, which provides continuous electrical pathways throughout the mat and unrestricted electrolyte percolation through the inter-fiber microporous voids [20,32].

Quantitative integration of the discharge curves yields a specific gravimetric capacitance of 274.5 F g^−1^ at 0.25 A g^−1^, which remains as high as 240.0 F g^−1^ at 2.5 A g^−1^, representing a remarkable 87% retention (Figure 6d). The large capacitance at low current density reflects the dominant contribution of micropore charge storage. In contrast, the moderate decrease in capacitance at higher current densities is mitigated by the fast ion-transport pathways provided by the 8–12 nm mesopores and the porous fibrous network. This behavior is consistent with the hierarchical pore structure generated through the sodium-assisted templating strategy, which provides extensive microporosity while preserving efficient ion transport pathways [32]. In absolute terms, the specific capacitance compares favorably with those reported for biomass-derived, free-standing lignin carbon nanofibers (Table 1) [20,32], highlighting the effectiveness of the proposed pore-engineering approach.

Long-term cycling tests further demonstrate the excellent reversibility and stability of the LCF-AW electrode (Figure 6e). The symmetric cell retained 99.9% coulombic efficiency after 10,000 cycles at 10 A g^−1^. After an initial activation period of approximately 100 cycles, the capacitance gradually increased and stabilized at about 117% of its initial value. This increase above 100% may be attributed to the progressive electrochemical activation of the electrode, which enhances electrolyte wetting and gradually improves access to initially less accessible pore regions, thereby increasing the electrochemically active surface area. The nearly identical triangular GCD profiles recorded after 1000 and 10,000 cycles (Figure 6e, inset) further demonstrate the outstanding electrochemical stability and the highly reversible electric double-layer charge-storage behavior of the hierarchical carbon nanofiber electrode. Given that charge storage is predominantly governed by electric double-layer capacitance rather than by intercalation or conversion reactions, the preservation of the characteristic GCD profiles suggests that no substantial changes in the carbon framework are induced during prolonged cycling.

The Ragone plot (Figure 6f) further highlights the excellent rate capability of the LCF-AW electrode, delivering a maximum energy density of 38.1 W h kg^−1^ at 125 W kg^−1^, retaining 33.3 W h kg^−1^ even at power densities exceeding 1000 W kg^−1^. This corresponds to an energy loss of just 12% despite nearly one order of magnitude increase in power density, indicating efficient utilization of the accessible pore network even under high-rate operation. These values compare favorably with those reported for other biomass- and lignin-derived free-standing carbon nanofiber electrodes evaluated under comparable two-electrode configurations (Table 1). More importantly, the results demonstrate that the proposed sodium-assisted in situ templating strategy enables the fabrication of free-standing carbon nanofiber electrodes combining high energy density, excellent rate capability, and outstanding cycling stability without the need for aggressive chemical activation [32].

## 4. Conclusions

A simple and scalable strategy has been developed to fabricate free-standing, binder-free carbon nanofiber electrodes from lignin/PEO through electrospinning in an aqueous NaOH medium followed by stabilization, carbonization, and mild acid washing. Rather than conventional chemical activation, the proposed route exploits the in situ formation and selective removal of sodium-rich phases to engineer a hierarchical micro–mesoporous architecture while preserving the structural integrity of the carbon nanofibers. The resulting materials exhibit predominantly microporous texture (84% of the specific surface area) combined with mesopores centered at 8–12 nm and a high specific surface area (from 74.4 to 776.8 m^2^ g^−1^).

The resulting carbon nanofiber electrodes combine high capacitance, excellent rate capability, and outstanding cycling stability, delivering a specific capacitance of 274.5 F g^−1^ while retaining 87% of this value at high current density. They also achieved a competitive energy density of 38.1 W h kg^−1^, maintaining 33.3 W h kg^−1^ at power densities above 1000 W kg^−1^, along with 99.9% coulombic efficiency and a capacitance retention of 117% after 10,000 charge–discharge cycles. These results show that electrochemical performance is primarily governed by engineered hierarchical pore architecture rather than by extensive heteroatom functionalization, providing valuable design principles for sustainable biomass-derived carbon electrodes.

Beyond the specific performance achieved for supercapacitors, this work establishes sodium-assisted in situ templating as a versatile strategy for tailoring hierarchical porosity in sustainable lignin-derived carbon fibers without aggressive activation treatments or hazardous etching agents. This strategy provides a low-cost and practical route for designing accessible porous carbons for electrochemical energy storage systems and other applications requiring tailored pore architectures, including adsorption, separation, and heterogeneous catalysis.

## Figures and Tables

**Table 1:** Comparison of the textural and electrochemical figures of merit of the NaOH-templated lignin carbon nanofibers developed in this work with representative carbon materials reported in the literature.

Material	Activator	SSA * (m^2^ g^−1^)	Cs (F g^−1^)	Electrolyte	Capacitance Retention (Cycles)	Ref.
PAN	PmAP	1031.4	347 (0.5 A g^−1^)	6 M KOH	90.5% (10,000)	[79]
ELH	Non-activated	675.0	217 (1 A g^−1^)	6 M KOH	88.8% (2000)	[80]
Lignin	TEOS	1197.0	282 (0.2 A g^−1^)	6 M KOH	91.5% (10,000)	[24]
ELH	KOH	2285.0	187 (0.5 A g^−1^)	6 M KOH	96.1% (10,000)	[21]
Wood biochar	Fluorine	472.0	407 (0.5 A g^−1^)	1 M H_2_SO_4_	91.0% (10,000)	[81]
LCF-AW	** NaOH	776.8	275 (0.25 A g^−1^)	3 M KOH	117.0% (10,000)	This work

* SSA = specific surface area; Cs = Specific capacitance; ELH = Enzymatic lignin hydrolysis; TEOS = Tetraethyl orthosilicate; PmAP = poly(m-aminophenol). ** NaOH is not used as an activator; it serves as an in situ pore-forming template.

## Data Availability

The original contributions presented in this study are included in the article/Supplementary Material. Further inquiries can be directed to the corresponding authors.

## Supplementary Materials

The following supporting information can be downloaded at: https://www.mdpi.com/article/10.3390/polym18182203/s1, Figure S1. Schematic representation of the electrospinning process and operating parameters used to prepare the lignin/PEO mats. (a), together with a photograph of the resulting as-spun lignin mat (b), SEM image of LF (c), Na EDX map (inset in c), and fiber-diameter distribution (d); Figure S2. XRD pattern of the acid-washed carbonized lignin fibers (LCF-AW) compared with the reference diffraction pattern of crystalline Na_2_CO_3_. The broad diffraction features of LCF-AW are characteristic of poorly ordered carbon, while no crystalline reflections attributable to Na_2_CO_3_ are detected after acid washing; Table S1. EDX of LF and LCF-AW; Table S2. FTIR spectra of lignin, PEO, urea, lignin fiber (LF), and carbonized fiber (LCF-AW); Table S3. DSC curves of lignin, PEO, urea, and LF; Table S4. TGA and DTG curves of lignin, PEO, urea, LF under N_2_, and LF-oven under air and N_2_; Figure S3. (a) and (b) display the deconvoluted high-resolution spectra of O 1s and N 1s, respectively; Table S5. Textural parameters of the lignin-derived carbon fibers before and after acid washing, including the urea-free control (LCF-U-free).
