# Supplementary material for: Microporous and Mesoporous Carbon Nanofibers from Lignin by In Situ Sodium Templating for Supercapacitor Electrodes
Source: Polymers (Basel). 2026 Sep 10;18(18):2203. doi: 10.3390/polym18182203 (PMC13611747; doi:10.3390/polym18182203)
Supplement: Supplementary file 1 [file polymers-18-02203-s001.zip › polymers-4499244-supplementary.pdf]

Article

Microporous and Mesoporous Carbon Nanofibers from Lignin by In Situ Sodium Templating for Supercapacitor Electrodes

Linet Hernández-Gil [1], Zhu Peng Shen [1], Ainhoa Álvarez-Gómez [1], María Fernández-Álvarez [2], Juan P. Fernández Blázquez [3], Juan C. Cabanelas [1], Verónica San-Miguel [1,*] and María B. Serrano [1,*]

[1] Department of Materials Science and Engineering and Chemical Engineering (IAAB), Universidad Carlos III de Madrid, Avda. de la Universidad 30, 28911 Leganes, Spain; khernand@ing.uc3m.es (L.H.-G.); zshen@ing.uc3m.es (Z.P.S.); ainhoa.alvarezg21@gmail.com (A.Á.); caba@ing.uc3m.es (J.C.C.)

[2] Instituto de Cerámica y Vidrio (ICV), CSIC, Kelsen 5, 28049 Madrid, Spain; maria.fernandez@icv.csic.es

[3] IMDEA Materials Institute, C/Eric Kandel 2, 28906 Getafe, Spain; juanpedro.fernandez@imdea.org

* Correspondence: vmiguel@ing.uc3m.es (V.S.-M.); berna@ing.uc3m.es (M.B.S.)

Supporting information

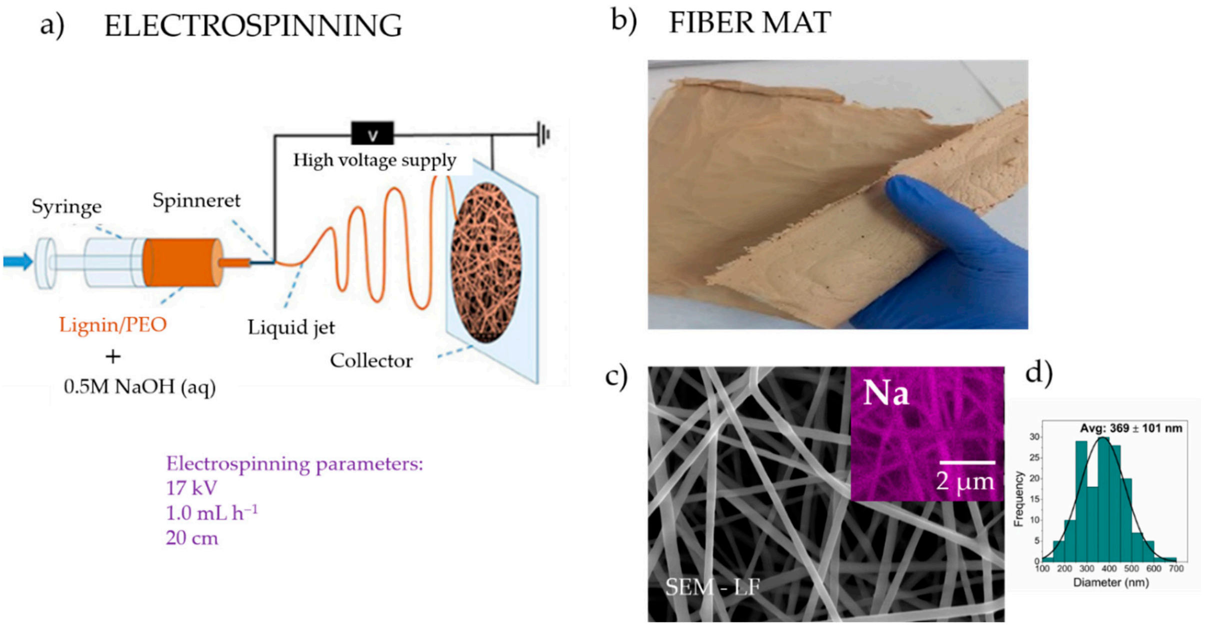

Figure S1. Schematic representation of the electrospinning process and operating parameters used to prepare the lignin/PEO mats. (a), together with a photograph of the resulting as-spun lignin mat (b), SEM image of LF (c), Na EDX map (inset in c), and fiber-diameter distribution (d).

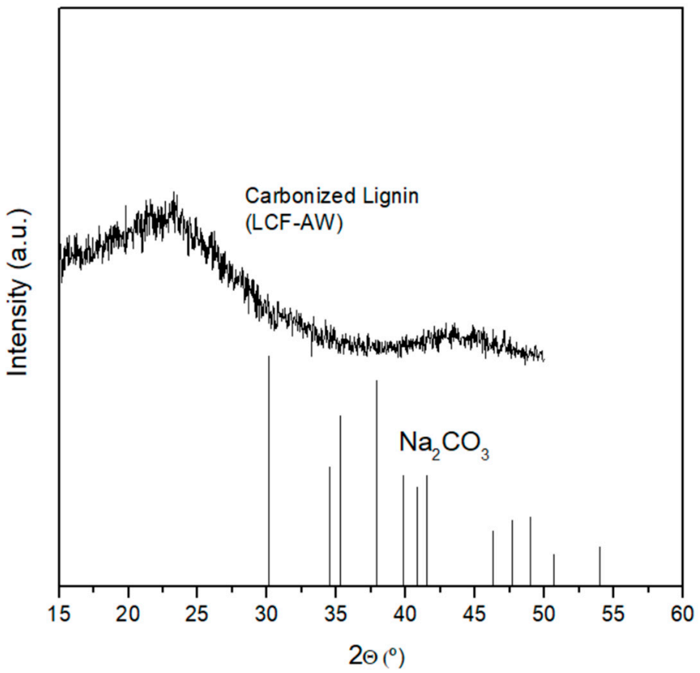

Figure S2. XRD pattern of the acid-washed carbonized lignin fibers (LCF-AW) compared with the reference diffraction pattern of crystalline Na_2CO_3. The broad diffraction features of LCF-AW are characteristic of poorly ordered carbon, while no crystalline reflections attributable to Na_2CO_3 are detected after acid washing.

Table S1. EDX of LF and LCF-AW.

Element	Atomic %	
	**LF**	**LCF-AW**
C K	62.59	84.77
O K	20.09	3.81
**N* K**	8.94	10.76
Na K	6.62	0.13

* EDX-derived nitrogen values are reported for completeness but are not considered quantitatively due to the limited reliability of EDX for nitrogen analysis in this carbon-rich matrix. Nitrogen surface content is instead evaluated by XPS.

Table S2. FTIR spectra of lignin, PEO, urea, lignin fiber (LF), and carbonized fiber (LCF-AW).

Sample	Band (cm^{-1})	Assignment
Lignin powder	3400 (broad)	O–H
	2900	C–H
	1600	C=C
	1200–1000	C–O
PEO powder	2880	C–H
	1100	C–O–C
	955 and 842	CH_2
Urea powder	3440–3340	N–H
	1606	N–H

	1457 and 1146	C–N
LF	3500–3300 and 1100	O–H, N–H, and C–O–C
LCF-AW	Almost complete disappearance of PEO, urea, and oxygen-containing lignin bands	

Table S3. DSC curves of lignin, PEO, urea, and LF.

Sample	Peak Temperature	Thermal event
PEO powder	70 °C	Tm
Urea powder	140 °C	Tm
Lignin powder	100 °C	Water evaporation
	149 °C	Tg
	300-400 °C	Thermal decomposition
LF	85 °C	Water evaporation (lignin)
	144 °C	Tm (PEO and Urea)

Table S4. TGA and DTG curves of lignin, PEO, urea, LF under N_2, and LF-oven under air and N_2.

Sample	Temperature range	DTG peak(s)	Residue at 800 °C
Lignin powder	110–800 °C	low intensity	46%
PEO powder	Stable to 317 °C	392 °C	~2%
Urea powder	Stable to 130 °C	-	Negligible
	190–370 °C	204, 243, 307, and 370 °C	
LF (N_2)	<120 °C	—	26%
	150–300 °C	—	
	350–500 °C	366 °C	
	>500 °C	—	
LF-oven (air 200 °C, then N_2)	<200 °C	-	~20%
	>200 °C	345 °C	

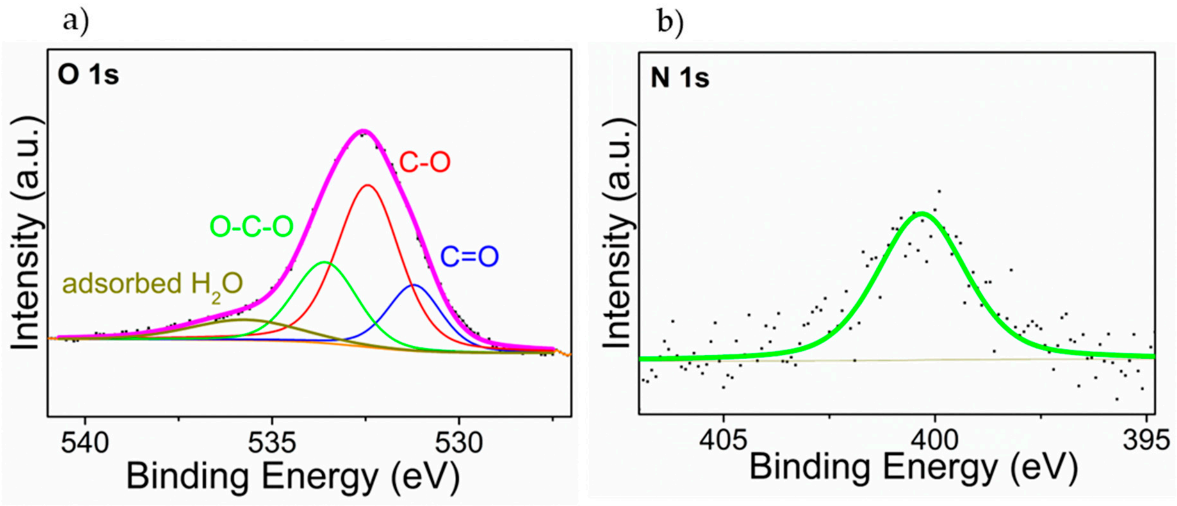

Figure S3. (a) and (b) display the deconvoluted high-resolution spectra of O 1s and N 1s, respectively.

Table S5. Textural parameters of the lignin-derived carbon fibers before and after acid washing, including the urea-free control (LCF-U-free).

Sample	BET specific surface ($m^2\ g^{-1}$)	Micropore area ($m^2\ g^{-1}$)	Micropore volume ($cm^3\ g^{-1}$)
**LCF** (carbonized fibers with urea, unwashed)	74.4	29.8	0.012
**LCF-U-free** (carbonized fibers urea-free control, unwashed)	119.8	52.3	0.025
**LCF-AW** (acid-washed carbonized fibers with urea)	776.8	654.2	0.250
